# Recent Findings in Physical Exercise for Cancer Survivors

**DOI:** 10.1298/ptr.R0023

**Published:** 2023-03-22

**Authors:** Shinichiro MORISHITA, Katsuyoshi SUZUKI, Taro OKAYAMA, Junichiro INOUE, Takashi TANAKA, Jiro NAKANO, Takuya FUKUSHIMA

**Affiliations:** ^1^Department of Physical Therapy, School of Health Sciences, Fukushima Medical University, Japan; ^2^Division of Rehabilitation Medicine, Shizuoka Cancer Center, Japan; ^3^Division of Rehabilitation Medicine, Kobe University Hospital International Clinical Cancer Research Center, Japan; ^4^Department of Rehabilitation, Hyogo Medical University Hospital, Japan; ^5^Faculty of Rehabilitation, Kansai Medical University, Japan

**Keywords:** Cancer survivor, Physical exercise, Complication, Prognosis

## Abstract

In recent years, the number of cancer survivors has been increasing each year due to advances in the early diagnosis and treatment of cancer. Cancer survivors present a variety of physical and psychological complications due to cancer and its treatment. Physical exercise is an effective nonpharmacological treatment for complications in cancer survivors. Furthermore, recent evidence has shown that physical exercise improves the prognosis of cancer survivors. The benefits of physical exercise have been widely reported, and guidelines for physical exercise for cancer survivors have been published. These guidelines recommend that cancer survivors engage in moderate- or vigorous-intensity aerobic exercises and/or resistance training. However, many cancer survivors have a poor commitment to physical exercise. In the future, it is necessary to promote physical exercise among cancer survivors through outpatient rehabilitation and community support.

**T**he number of cancer cases is increasing every year. It is estimated that 19.3 million people worldwide were diagnosed with cancer in 2020^1)^ and 28.4 million people will be newly diagnosed in 2040^1)^. In Japan, there were approximately 1,012,000 new cancer cases in 2020^2)^. In recent years, advances in cancer screening and treatment have led to improved survival rates and an increase in the number of cancer survivors^2,3)^. Furthermore, about half of the growing number of cancer survivors are elderly adults^2)^. The number of elderly cancer survivors will continue to grow as the population ages.

## Complications for Cancer Survivors

**Fig. 1.**
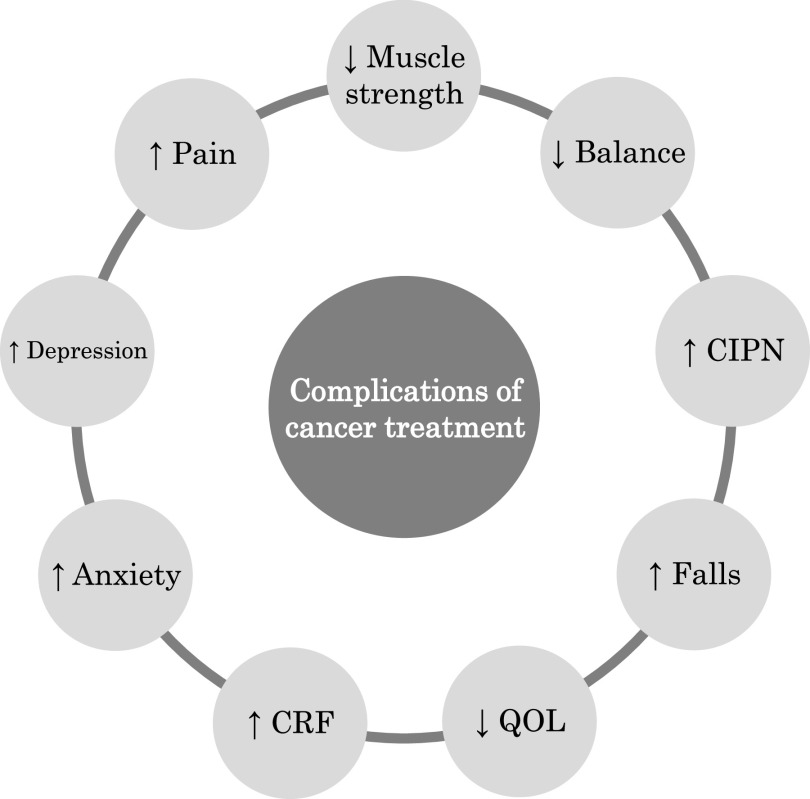
Various complications of cancer treatment in cancer survivors

CIPN, chemotherapy-induced peripheral neuropathy; QOL, quality of life; CRF, cancer-related fatigue

Cancer treatment is selected based on the type of tumor, site, stage, and pathological diagnosis. In addition, cancer treatment can affect nontarget tissues^4)^ and cancer cells also adversely affect bodily functions^5)^. Therefore, cancer survivors experience various complications due to the effects of cancer and its treatment (Fig. 1).

A previous study has reported breast cancer survivors to have lower upper body muscle strength than healthy controls^6)^. Another study reported that 26 cancer survivors had significantly lower hand grip strength than age-matched healthy controls^7)^. These reports indicate that cancer survivors may have reduced muscle strength compared to healthy controls.

Cancer survivors may also have impaired balance function. In a study by Schmitt et al., cancer survivors exhibited longer mediolateral root mean square and increased center of pressure velocity compared to healthy controls^8)^. Another previous study reported that cancer survivors had lower Mini-Balance Evaluation Systems Test scores and longer Timed Up and Go test times compared to healthy controls^7,9)^. With eyes open, the area of the center of pressure was significantly larger in cancer survivors than in healthy controls^9)^. These findings indicate that cancer survivors have impaired balance function compared to healthy individuals. This lower balance function in cancer survivors may be related to chemotherapy-induced peripheral neuropathy (CIPN). CIPN presents primarily with sensory and motor neuropathy in the hands and feet^10)^. A meta-analysis of 31 studies of 4179 cancer survivors showed that the prevalence of CIPN is 68.1% at one month, 60.0% at three months, and 30.0% at six months after chemotherapy^11)^. CIPN also negatively affects physical functions such as upper and lower extremity function^12,13)^, gait^12,14)^, and balance function^14,15)^, and is one of the main concerns of cancer survivors.

Cancer survivors present with various physical symptoms, including pain, fatigue, and anxiety. The prevalence of pain in cancer survivors is 39.3% after curative treatment, 55.0% during anticancer therapy, and 66.4% during advanced/metastatic/terminally ill stages^16)^. Cancer-related fatigue (CRF) is prevalent among cancer survivors and may continue to be experienced long after treatment^17)^. Additionally, a study of the prevalence of anxiety and depression in 1154 adult cancer survivors showed that at six months post diagnosis, the prevalence of anxiety and depression was 22% and 13%, respectively, and then 21% and 13%, respectively, at 12 months^18)^.

Cancer survivors’ complications negatively affect their quality of life (QOL). Previous studies have shown that cancer survivors have a lower QOL than healthy controls^19)^, and there is a significant association between muscle strength and QOL^19)^. Kober et al. reported that cancer survivors with peripheral neuropathy had poorer balance function and lower QOL scores than those without peripheral neuropathy, particularly in the physical function domain^13)^. On the other hand, another study reported that balance function in cancer survivors might have little effect on QOL^20)^. Therefore, cancer survivors’ muscle strength may more significantly impact their QOL than their balance function.

Elderly cancer survivors, as well as adult cancer survivors, present a variety of complications. In a previous study, 3766 elderly endometrial cancer survivors had more difficulty with walking and/or balance than an age- and race-matched group of women with no cancer history^21)^. Another report showed that elderly breast cancer survivors had lower short physical performance battery scores, longer chair stand times, and lower grip strength than women of the same age without cancer^22)^. Thus, elderly cancer survivors may have poorer physical function than healthy elderly adults.

Falls are a significant problem for elderly cancer survivors. A systematic review of falls in elderly cancer survivors reported a fall rate of 1.52%–3.41% per 1000 patient days for inpatients and 39%–64% for outpatients^23)^. Furthermore, falls in elderly cancer survivors have been shown to impact subsequent cancer treatment negatively^23)^.

In summary, adult cancer survivors present with various complications and reduced QOL due to cancer and its treatment. In addition, elderly cancer survivors have poor physical function and a higher risk of falls.

## Effects of Physical Exercise on Complications

**Fig. 2.**
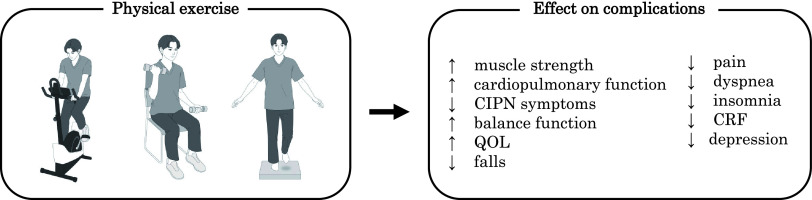
Effects of physical exercise on complications in cancer survivors

CIPN, chemotherapy-induced peripheral neuropathy; QOL, quality of life; CRF, cancer-related fatigue

Physical exercise is an effective nonpharmacological treatment for complications in cancer survivors (Fig. 2). A systematic review of 16 randomized controlled trials (RCTs) of cancer survivors undergoing active treatment showed that physical exercise, including aerobic exercise and/or resistance training, improved muscle strength more than usual care^24)^. Another meta-analysis, including 48 RCTs with cancer survivors (n = 3632), showed that physical exercise significantly improved cardiopulmonary function^25)^. In that study, 56% of physical exercise was aerobic^25)^.

The effectiveness of physical exercise for CIPN has also been evaluated. A systematic review and meta-analysis of five RCTs found that physical exercise significantly improved mean CIPN scores^26)^. The physical exercises included resistance, balance, sensorimotor-based, and nerve gliding exercises^26)^. In contrast, another meta-analysis found no effect of physical exercise on CIPN symptoms^27)^. That study showed that physical exercise significantly positively affected physical function (muscle strength, balance function) and QOL in cancer survivors with CIPN^27)^.

The effects of physical exercise on physical symptoms in cancer survivors have been investigated. A meta-analysis of 10 RCTs found that physical exercise significantly improved physical symptoms such as fatigue, pain, dyspnea, and insomnia in cancer survivors compared to a healthy control group^28)^. The types of physical exercise included aerobic, resistance, stretching, and walking^28)^. A meta-analysis on the effects of pharmacotherapy, psychotherapy, and physical exercise on CRF was conducted^29)^, and showed that physical exercise significantly improved CRF compared to other treatments^29)^. In studies of physical exercise (n = 69), 36 involved aerobic exercises, 13 involved anaerobic exercises, and 20 involved a combination of aerobic and anaerobic exercises^29)^. Another meta-analysis of 37 RCTs of 2929 cancer survivors showed that physical exercise reduced depressive symptoms compared to standard treatment^30)^. Exercise modalities included walking, yoga, stationary cycling, resistance bands, and weight machines^30)^.

Several studies have reported that physical exercise is an effective intervention for improving the QOL of cancer survivors. A meta-analysis of 16 RCTs showed that physical exercise significantly improved the QOL of cancer survivors compared to usual care^31)^. Outcomes associated with QOL, such as fatigue and physical function, were also reported to improve with physical exercise^31)^. In addition, another meta-analysis showed that physical exercise for cancer survivors during and after treatment significantly improved QOL and physical function compared to that for the control group^32)^. Among the forms of exercise delivery in that study, supervised physical exercise was shown to have the largest positive impact on QOL and physical function^32)^. Another study investigated the effects of physical exercise on various domains of cancer survivors’ QOL^33)^, and the results showed that physical exercise improved global QOL, physical QOL, role QOL, and emotional QOL^33)^.

There is a lack of evidence on the effect of physical exercise on falls, a concern in elderly cancer survivors^34)^. Currently, trials of physical exercise interventions are being tested in elderly cancer survivors to assess their ability to prevent falls^35)^.

In summary, many studies have suggested that physical exercise may improve muscle strength, cardiopulmonary function, CIPN symptoms, balance function, pain, dyspnea, insomnia, CRF, depression, and QOL in cancer survivors. Further research is needed on the effects of physical exercise on falls.

## Prognostic Impact of Physical Exercise

**Fig. 3.**
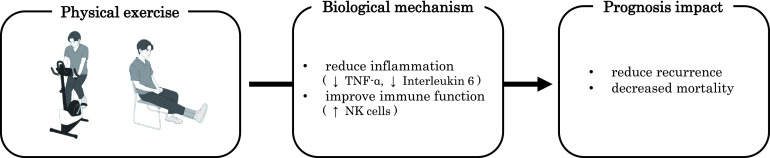
Prognostic impact of physical exercise

Physical exercise has been shown to improve prognosis in cancer survivors (Fig. 3). Studies in the 2000s showed that physical activity improved the prognosis of breast and colorectal cancer survivors. For example, participation in physical activity after diagnosis reduced recurrence by 24% and mortality by 45% in breast cancer survivors^36)^. Similarly, recurrence and mortality were reduced by 40% and 63%, respectively, among colorectal cancer survivors^37)^.

TNF-α, tumor necrosis factor-α; NK, natural killer

Since the 2010s, more systematic reviews and meta-analyses have been reported. A meta-analysis of 136 studies found evidence that cancer survivors with higher physical activity levels after diagnosis had reduced cancer-specific mortality^38)^. In addition, a meta-analysis of RCTs on physical exercise among cancer survivors has been investigated^39)^. Eight RCTs were included in the meta-analysis, and interventions were roughly classified into 2 categories: short-term interventions in the hospital (two weeks in duration) and outpatient or home-based interventions (2–8 months in duration)^9)^. All physical exercises were aerobic and/or resistance training^39)^. A meta-analysis revealed that physical exercise reduced cancer survivors’ risk of recurrence by about 48% and the risk of mortality by about 24%^39)^.

The biological mechanisms for the prognostic effects of physical exercise have not yet been established. In 2016, the antitumor effects of physical exercise were demonstrated in animal studies^40)^. In a mouse model, six weeks of physical exercise reduced the incidence and growth of several tumors (melanoma, liver cancer, and lung cancer)^40)^. In addition, physical exercise was shown to regulate tumor growth by increasing the number of natural killer (NK) cells^40)^. The mobilization of NK cells involved interleukin-6, released from muscles as a result of physical exercise^40)^.

Studies of cancer survivors suggest that physical exercise may maintain NK cell function^41)^. However, there is currently limited evidence to support the effects of physical exercise on NK cells in cancer survivors^42)^. On the other hand, evidence for an anti-inflammatory effect of physical exercise in cancer survivors is becoming more evident^43)^. A meta-analysis of 26 RCTs showed that physical exercise reduces inflammatory markers, particularly C-reactive protein levels and tumor necrosis factor-α production^43)^. The anti-inflammatory effects of physical exercise were more pronounced in prostate and breast cancer survivors^43)^, and the most effective physical exercise program was a combination of aerobic and resistance training^43)^.

In summary, physical exercise has been shown to influence the prognosis of cancer survivors. Recently, meta-analyses using RCTs, not only cohort studies, have been conducted, and the evidence in this area is growing. On the other hand, the biological mechanisms of the exercise effect have not been established and require further study. Cancer recurrence is a significant concern for most cancer survivors^44)^. Therefore, physical therapists need to provide physical exercise to improve the prognosis of cancer survivors in the future.

## Physical Exercise Guidelines for Cancer Survivors

**Fig. 4.**
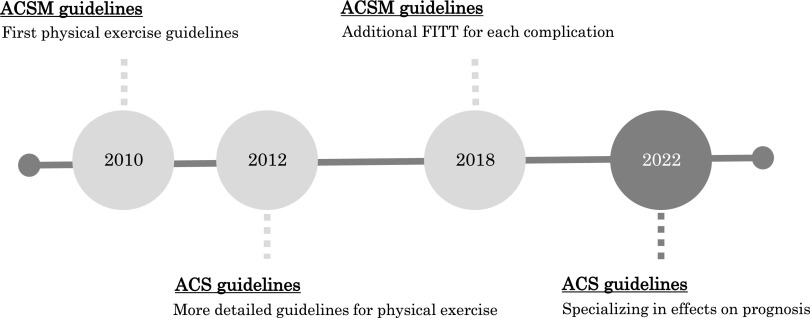
History of physical exercise guidelines for cancer survivors

ACSM, American College of Sports Medicine; FITT, frequency, intensity, time, and type; ACS, American Cancer Society

The American Cancer Society (ACS) and the American College of Sports Medicine (ACSM) have published recommendations on physical exercise for cancer survivors^45–49)^. This chapter presents several guidelines (Fig. 4).

In 2010, the ACSM developed the first physical exercise guidelines for cancer survivors^46)^. These guidelines reported strong evidence that physical exercise improves physical fitness, physical functioning, QOL, and CRF. However, there was insufficient evidence to establish a recommended amount of physical exercise. Therefore, based on the Physical Activity Guidelines for Americans, the guidelines recommend at least 150 minutes of aerobic exercise per week and resistance training at least two days per week.

In 2012, the ACS published more detailed recommendations for physical exercise^47)^. Adult cancer survivors are recommended to perform 150 minutes per week of aerobic exercise at a moderate intensity or 75 minutes per week at a vigorous intensity, plus resistance training for major muscle groups at least two days per week. Elderly cancer survivors should follow the recommendations of adult cancer survivors if possible, but if there are limitations, it is recommended that they exercise whenever possible and avoid prolonged physical inactivity.

In 2019, the ACSM released new physical exercise recommendations for cancer survivors^48)^. These guidelines provided recommendations and frequency, intensity, time, and type (FITT) for various complications in cancer survivors. Specifically, they reported strong evidence to support the effectiveness of physical exercise for anxiety, depressive symptoms, CRF, health-related QOL, lymphedema, and physical function. The guidelines also provided moderate evidence for bone health and sleep and insufficient evidence for cardiotoxicity, CIPN, cognitive function, falls, nausea, pain, sexual function, and treatment tolerance. For FITT, the introductions focused mainly on outcomes in the strong evidence category. For most, 30 minutes of aerobic exercise three times a week is an effective exercise prescription. Resistance training, which consists of two or more sets of 8–15 repetitions at 60% or more of the maximum number of repetitions per session at least twice a week, has also been equally effective. Because cancer survivors have sequelae and comorbidities, individualized goals are recommended.

In 2022, the ACS published the latest recommendations for physical exercise^49)^. The purpose of these ACS guidelines was to provide recommendations for physical exercise (including nutrition) to improve prognosis. For physical exercise, the studies included systematic reviews, meta-analyses, cohort studies, and RCTs (sample sizes of at least 200 participants) published in 2018 or later. The review revealed that nine studies met the inclusion criteria and that physical exercise is recommended for breast, colorectal, and prostate cancer survivors to improve prognosis. There is currently limited evidence for the prognostic impact of physical exercise on survivors of gynecologic, lung, hematologic, and pediatric cancers. In addition, these guidelines recommend 150–300 minutes per week for adult cancer survivors at moderate intensity, 75–150 minutes of physical exercise at vigorous intensity, and resistance training at least two days per week. Children and adolescents should perform at least one hour of moderate- or vigorous-intensity physical exercise daily. Compared to the 2012 ACS guidelines, the recommended amount of physical exercise has been increased, and recommendations for cancer survivors in the adolescent and young adult generation have been added.

In summary, the guidelines for physical exercise for cancer survivors recommend that physical exercise should include aerobic exercise, resistance training, or a combination of both. In recent years, the guidelines have also shown that physical exercise can improve the prognosis of cancer survivors. Recommended physical exercise is provided, but cancer survivors have various complications and should be evaluated individually.

## Future Directions

Physical exercise is a critical nonpharmacological therapy to improve complications and prognosis in cancer survivors. However, most cancer survivors lack compliance with physical exercise^50–52)^. A previous study reported that in 102 cancer survivors, the amount of physical exercise significantly decreased after cancer diagnosis and remained decreased during treatment^50)^. Another report indicated that only 38% of 72 cancer survivors were able to meet 90 to 150 minutes of moderate to vigorous exercise per week and only 10% were able to perform resistance training twice per week^51)^. The amount of physical exercise over ten years for 631 breast cancer survivors was also studied, and the results showed that 7.8% met the guidelines for physical exercise at all follow-up periods^52)^. Thus, many cancer survivors lack compliance with physical exercise.

ACSM guidelines recommend supervised physical exercise for cancer survivors^48)^. However, in Japan, outpatient rehabilitation for cancer survivors is not covered by medical fees, and the implementation rate of outpatient rehabilitation is 39.1%^53)^. Therefore, a medical fee revision is needed to promote outpatient cancer rehabilitation.

Elderly cancer survivors have limited mobility^54)^. Therefore, community support is essential for cancer survivors. However, only 39.1% of the hospitals in Japan designated as cooperative cancer treatment centers have regional cooperation^53)^, and only 9.8% have established a regional cooperation path^53)^. In the future, regional cooperation should be promoted to support cancer survivors regarding physical exercise.

## Conclusions

Cancer survivors present not only physical complications, such as low muscle strength and balance function, but also psychological complications, such as anxiety, depression, and CRF, as a result of cancer and its treatment. Furthermore, these complications negatively affect QOL. Elderly cancer survivors likewise present a variety of complications, and falls are a significant problem. Physical exercise can improve physical and psychological complications in cancer survivors. Recently, physical exercise has even been shown to improve the prognosis of cancer survivors. The ACSM and ACS guidelines for cancer survivors recommend physical exercises such as aerobic exercise and resistance training to improve complications and prognosis. However, most cancer survivors lack compliance with physical exercise. To promote physical exercise among cancer survivors, inpatient rehabilitation alone is insufficient. In the future, outpatient rehabilitation and community support should be used to promote physical exercise among cancer survivors after discharge.

## Conflict of Interest

The authors declare no conflict of interest.
